# HBeAg-induced miR-106b promotes cell growth by targeting the retinoblastoma gene

**DOI:** 10.1038/s41598-017-14652-x

**Published:** 2017-10-30

**Authors:** Jasmine Samal, Manish Kandpal, Perumal Vivekanandan

**Affiliations:** Kusuma School of Biological Sciences, Indian Institute of Technology Delhi, New Delhi, India

## Abstract

Chronic HBV infection is a major cause of hepatocellular carcinoma (HCC). The association between hepatitis B “e” antigen (HBeAg) and HCC is well-established by epidemiological studies. Nonetheless, the biological role of HBeAg in HCC remains enigmatic. We investigate the role of HBeAg in HBV-related HCC. Our findings suggest that HBeAg enhances cell proliferation and accelerates progression from G0/G1 phase to the S phase of the cell cycle in Huh7 cells. Examination of host gene expression and miRNA expression profiles reveals a total of 21 host genes and 12 host miRNAs that were differentially regulated in cells expressing HBeAg. Importantly, HBeAg induced the expression of miR-106b, an oncogenic miRNA. Interestingly, HBeAg-expression results in a significant reduction in the expression of retinoblastoma (Rb) gene, an experimentally validated target of miR-106b. Inhibition of miR-106b significantly increased the expression of the Rb gene, resulting in reduced cell proliferation and slowing of cell cycle progression from the G0/G1 phase to S phase. These observations suggest that the up-regulation of miR-106b by HBeAg contributes to the pathogenesis of HBV-related HCC by down-regulating the Rb gene. Our results highlight a role for HBeAg in HCC and provide a novel perspective on the molecular mechanisms underlying HBV-related HCC.

## Introduction

Hepatitis B infection is a global health problem affecting more than 2 billion people worldwide. Hepatitis B infection can cause a wide spectrum of diseases ranging from acute HBV infection to chronic hepatitis B, cirrhosis and hepatocellular carcinoma (HCC). The persistence of hepatitis B e antigen (HBeAg) is associated with an increased risk of cirrhosis and HCC in patients with chronic hepatitis B (CHB)^1^. HBeAg, a secretory protein of hepatitis B virus (HBV), produced from the pre-C/C ORF (precore/core open reading frame) is normally detected in the serum of infected individuals when the virus is actively replicating^2,3^. The presence of HBeAg is a well-documented risk factor for HCC in epidemiological studies^4^. Importantly, the presence of HBeAg increases the risk of progression to HCC independent of virus loads^4^. The most common and clinically relevant mutation in HBV pre-C/C ORF leading to the loss of HBeAg is a G to A substitution at nucleotide 1896 (G1896A, resulting in a stop codon) leading to premature termination of translation of HBeAg^5^. The G1896A variant is associated with lower virus loads as compared to the HBeAg producing wild-type HBV^6^. Moreover, seroconversion from HBeAg to anti-HBe (antibody to HBeAg) during the course of CHB infection leads to better clinical outcomes^6,7^. However, the biological role of HBeAg in the pathogenesis of chronic HBV infection remains unknown. Most HBV-related HCC studies have investigated the function of HBx in regulating  the pathogenesis of liver cancer, as HBx is a transcriptional transactivator^8–10^. Apart from the HBx protein, the role of other HBV proteins in the pathogenesis of HBV-related HCCs remain poorly understood.

In this study, we aimed to investigate the role of HBeAg, if any, in HBV-related HCC. Our findings show that HBeAg enhances cell proliferation by accelerating G1/S phase transition in Huh7 cells. To understand the role of HBeAg in modulating cell cycle progression, we analyzed HBeAg-induced changes in host miRNA- and gene expression-profiles using microarrays. Importantly, we found that the presence of HBeAg induces miR-106b expression leading to a significant reduction in the expression of the retinoblastoma (Rb) gene. In addition, inhibition of miR-106b increased Rb expression and promoted accumulation of cells in G0/G1 phase of cell cycle, thus attenuating cell proliferation. Our results reveal a possible molecular mechanism that links HBeAg to the pathogenesis of HBV-related HCC.

## Results

### HBeAg promotes cell proliferation

**Figure 1:**
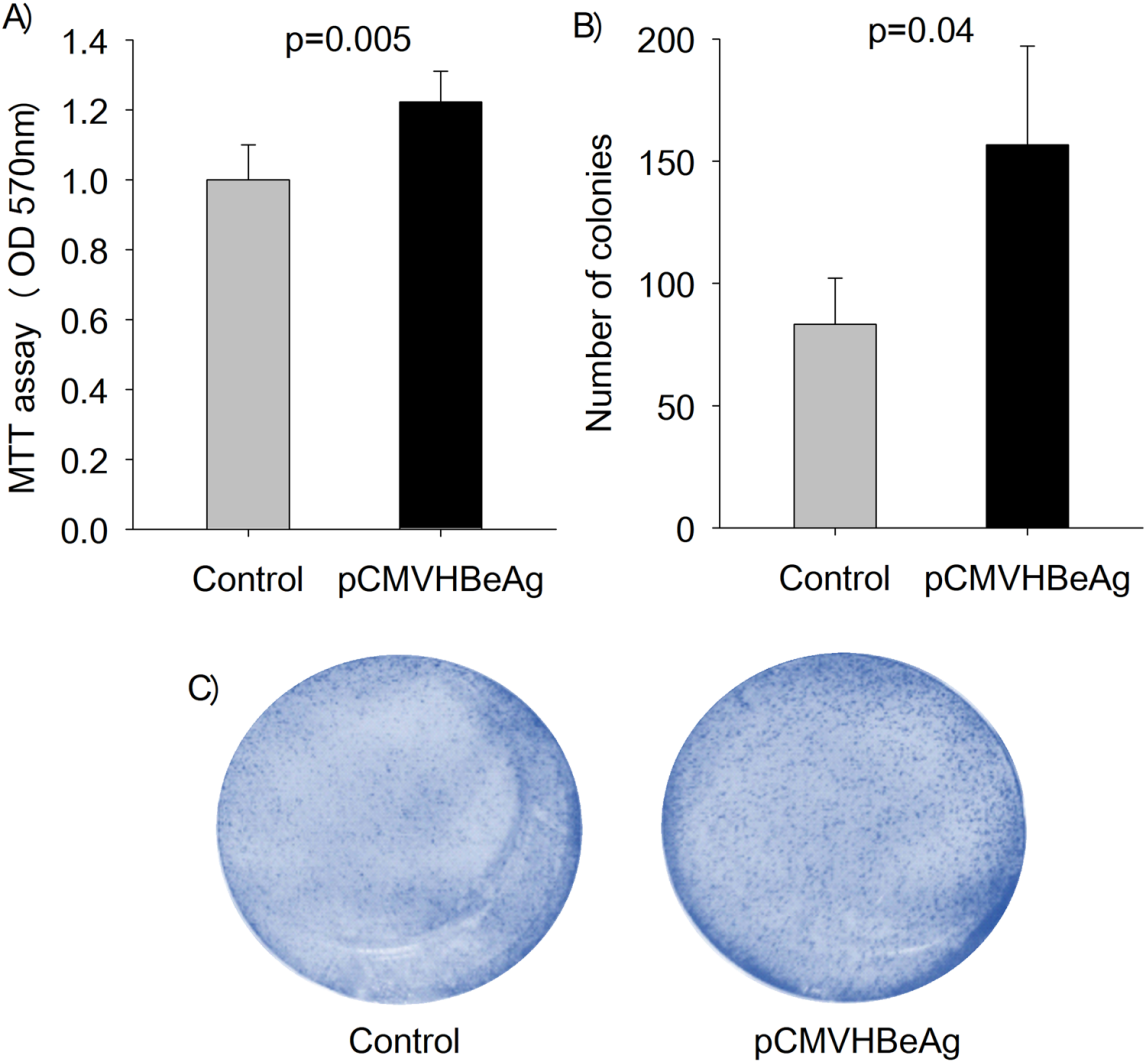
The presence of HBeAg is associated with increased cell proliferation. (**A**) Transient expression of HBeAg (pCMVHBeAg) in Huh7 cells results in enhanced cell proliferation as compared to that in the control (no HBeAg). (**B**) and (**C**) Transient expression of HBeAg (pCMVHBeAg) significantly increased colony formation in Huh7 cells as compared to that in the control (the bar graphs are represented as mean ± SD with n = 3).

The effect of HBeAg expression on cell proliferation was assessed using the MTT [3-(4,5-Dimethylthiazol-2-yl)-2,5-Diphenyltetrazolium Bromide] assay and colony formation assay. Interestingly, HBeAg promotes cell proliferation as measured by the MTT assay (Fig. 1A) and colony formation assay (Fig. 1B and C).

### HBeAg promotes G1/S transition in Huh7 cells

**Figure 2:**
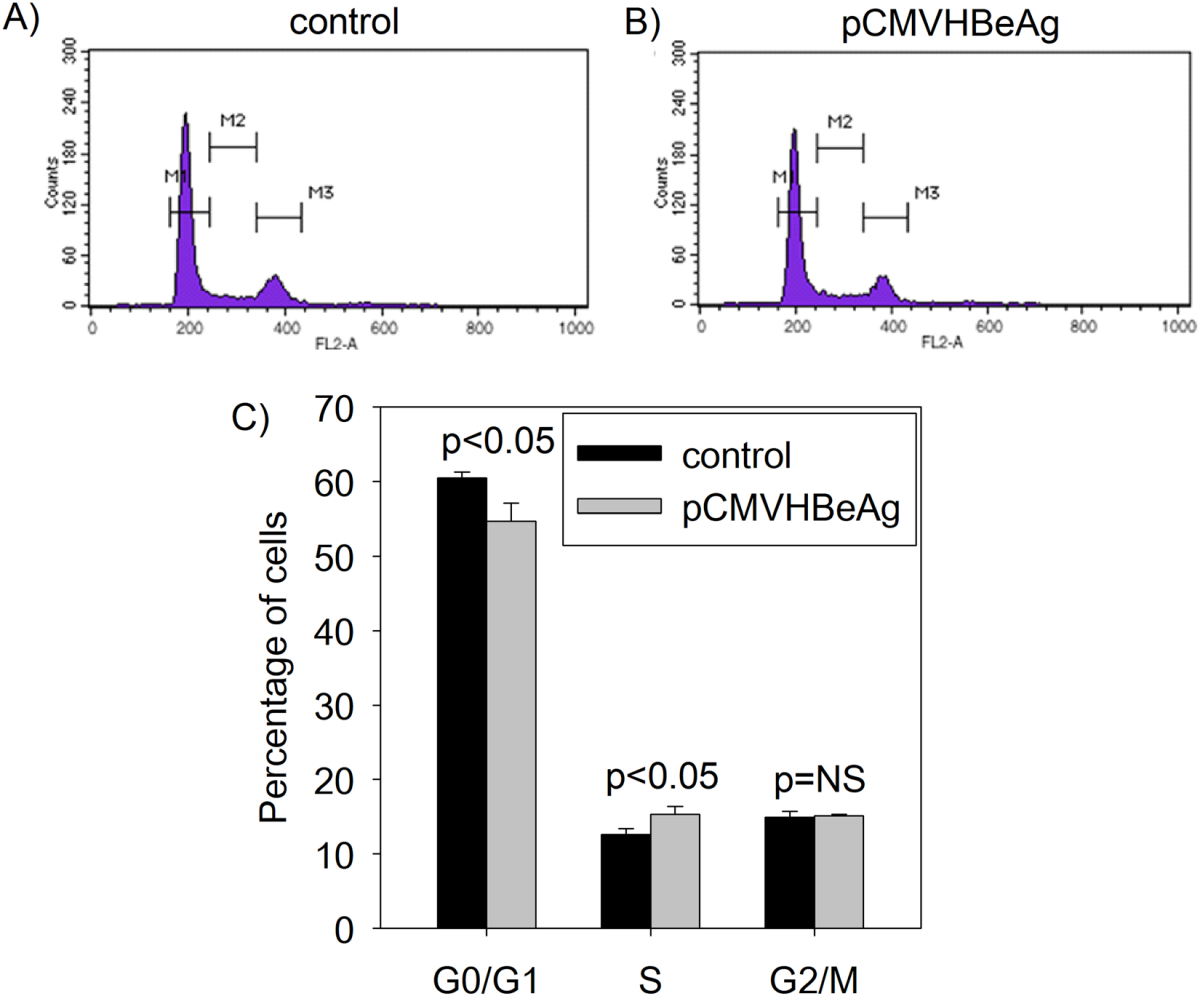
Effect of HBeAg expression on cell cycle profile in Huh7 cells using flow cytometry. Huh7 cells transfected with the (**A**) control (no HBeAg) or (**B**) pCMVHBeAg were analysed by PI staining and flow cytometry (The M1 peak corresponds to cells in G0/G1, M3 peak corresponds to cells in G2/M and cells in S phase are shown as M2). (**C**) A bar graph showing the proportion of cells in different phases of cell cycle in the presence (pCMVHBeAg) and absence (control) of HBeAg as analysed by flow cytometry (the bar graphs are represented as mean ± SD with n = 3; NS = not significant).

As cell proliferation is linked to cell cycle regulation, we investigated the effect of HBeAg expression on cell cycle progression using flow cytometry analysis. Strikingly, the presence of HBeAg in Huh7 cells results in decreased number of cells in the G0/G1 phase and increased number of cells in the S phase as compared to that in the control (Fig. 2). Our data suggest that HBeAg promotes G1/S transition in Huh7 cells.

### Analysis of HBeAg-induced dysregulation of host genes using cDNA microarray

**Figure 3:**
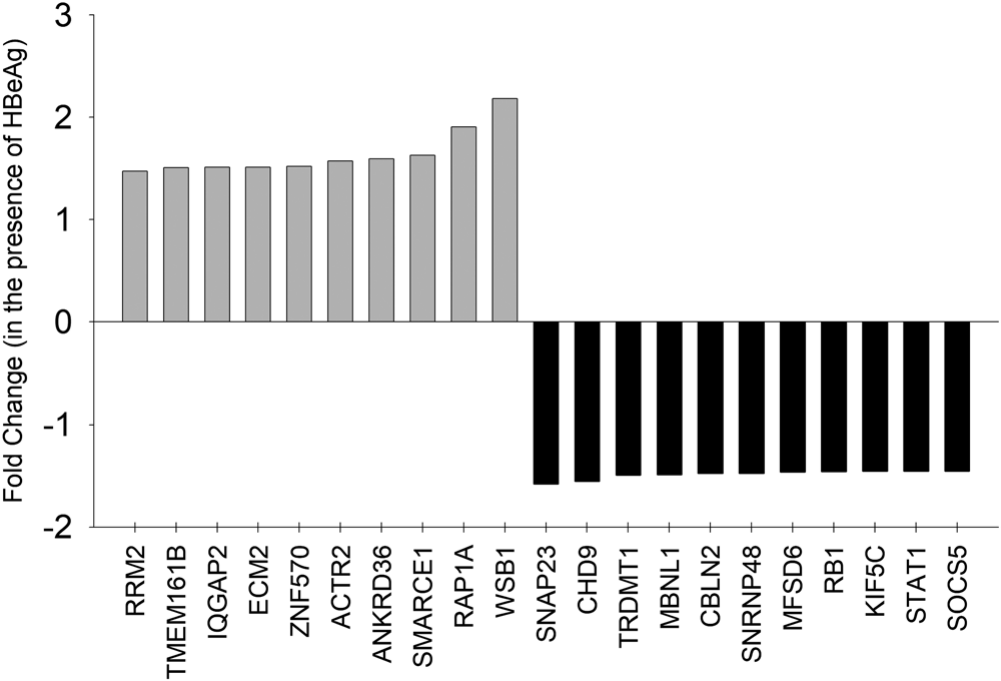
Identification of host genes differentially expressed in the presence of HBeAg in Huh7 cells using microarray analysis: A Bar graph showing host genes that are significantly up-regulated (light grey bars above the axis) and down-regulated (black bars below the axis) by HBeAg in Huh7 cells.

To better understand the mechanisms underlying the ability of HBeAg to modulate cell proliferation and cell cycle, we identified host genes which are differentially expressed in the presence of HBeAg using cDNA microarrays. A total of 21 genes were differentially expressed in the presence of HBeAg (as shown in Fig. 3). Ten genes were upregulated and eleven genes were down-regulated (fold change ≥ 1.5, p < 0.05) in the presence of HBeAg. The raw data files of the gene expression microarray experiments have been submitted to Gene Expression Omnibus (GEO) public database at the National Center for Biotechnology Information (NCBI) with accession number GSE90115.

### Identification of differentially expressed miRNAs in the presence of HBeAg

**Figure 4:**
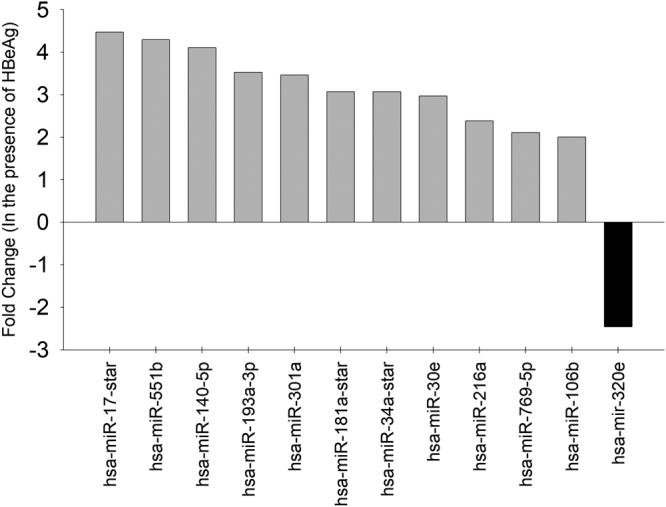
Identification of host miRNAs differentially expressed in the presence of HBeAg in Huh7 cells using microarray analysis: A Bar graph showing host miRNA that are significantly up-regulated (light grey bars above the axis) and down-regulated (black bars below the axis) by HBeAg in Huh7 cells.

Several lines of evidence suggest that dysregulation in host gene and microRNA (miRNA) expression profiles can contribute to the pathogenesis of cancer. Several groups have studied the role of miRNAs in the pathogenesis of HBV infection. Notably, recent studies have reported differences in the host miRNA expression between HBeAg-positive and HBeAg-negative CHB patients^11,12^. Intrigued by these recent reports and our findings on the role of HBeAg in cell proliferation/cell cycle progression and HBeAg-mediated dysregulation of host gene expression we chose to investigate the dysregulation of host miRNA by HBeAg, if any. Using miRNA microarrays, we identified a total of 12 miRNAs which were differentially expressed (fold change ≥ 2, p < 0.05) in the presence of HBeAg with 10 miRNAs upregulated and 1 miRNA downregulated (Fig. 4). The raw data files of the miRNA microarray experiments have been submitted to Gene Expression Omnibus (GEO) public database at the NCBI with accession number GSE90147.

### Integration of data from cDNA microarrays and miRNA microarrays

Integration of gene expression data and miRNA data is being increasingly used to identify putative mRNA targets and functional roles of miRNAs^13^. This approach may be particularly useful for our data since we have only a limited number of hits for both gene expression and miRNA expression.

To analyze if any of the differentially expressed genes (n = 21) is an experimentally validated target of any of the differentially expressed miRNA (n = 12), we used miRWalk 2.0 (a freely available webtool). We identified that miR-106b (a miRNA differentially expressed in the presence of HBeAg) and the Rb gene (a tumor suppressor gene differentially expressed in the presence of HBeAg) represent an experimentally validated miRNA-target pair. We then focused on miR-106b and the Rb gene.

### Validation of HBeAg-induced differential expression of miR106b and the Rb gene

**Figure 5:**
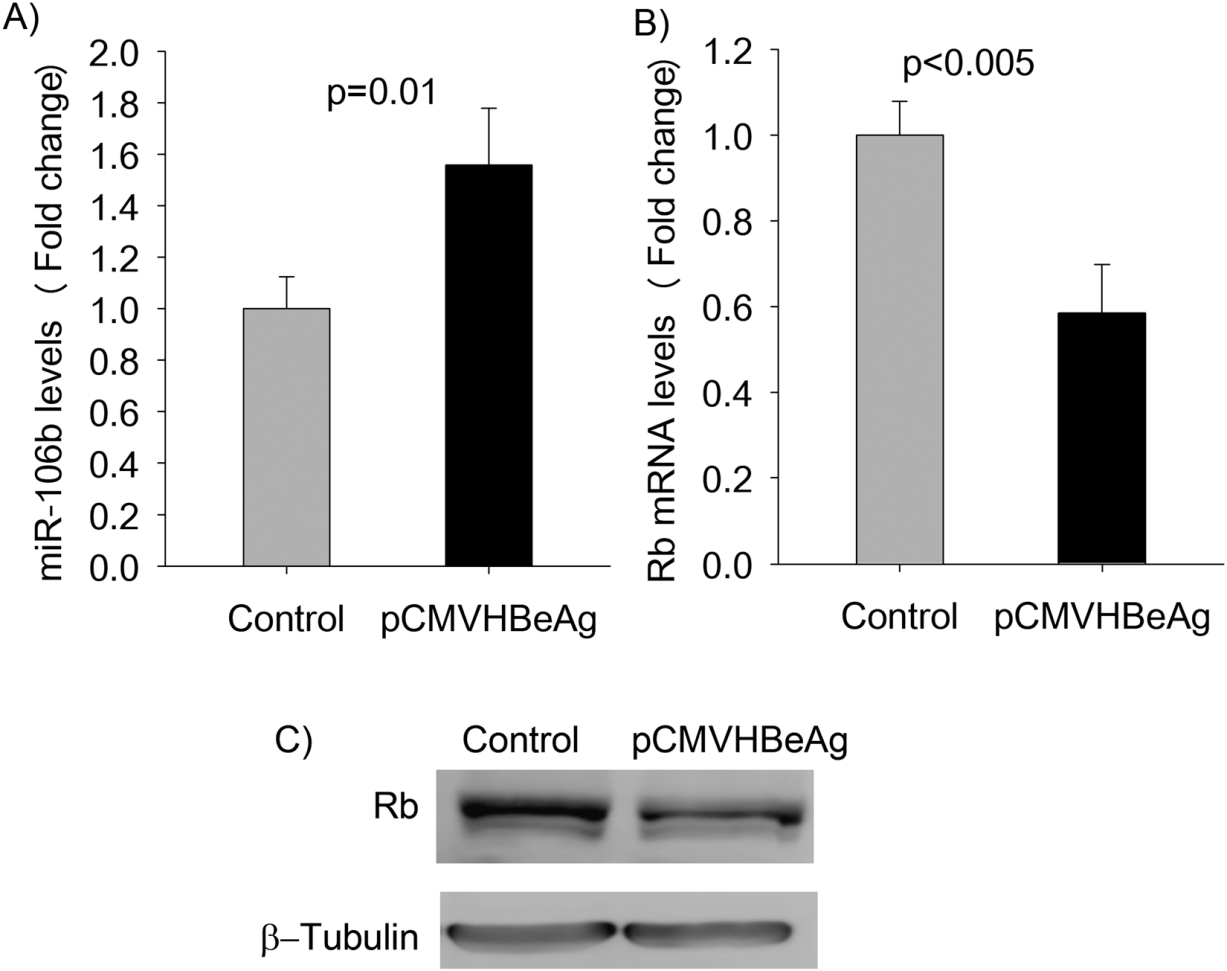
Validation of microarray findings on miR-106b and the Rb gene. (**A**) Quantitation of miR-106b expression levels in cells transfected with pCMVHBeAg (expresses HBeAg) or the control (no HBeAg) using real time PCR. (**B**) Real time PCR quantitation of Rb mRNA levels (normalized using GAPDH levels) in cells expressing HBeAg (pCMVHBeAg) and in control cells (transfected with the control plasmid). (**C**) Western Blot analysis of pRb expression in Huh7 cells (The full-length western blots images are shown in supplementary Fig. S2; the bar graphs are represented as mean ± SD with n = 3).

Real-time PCR results for miR-106b confirmed miRNA microarray findings that transient expression of HBeAg results in the up-regulation of miR-106b (Fig. 5A). Real-time PCR analysis validated the HBeAg-induced down-regulation of the Rb gene (Fig. 5B). This prompted us to further investigate the possible effect of HBeAg in HepG2, another liver cell line. Interestingly, HBeAg increased miR-106b levels by about 2.5 fold in HepG2 cells (supplementary Fig. S4). In addition, other HBV proteins including the HBV surface protein and HBx protein did not induce the expression of miR106b in Huh7 cells (supplementary Fig. S4). We then compared the retinoblastoma protein (pRb) expression between Huh7 cells transiently expressing HBeAg and control Huh7 cells (transfected with the control plasmid). In keeping with our results of lower Rb mRNA levels in the presence of HBeAg, pRb levels were also lower in the presence of HBeAg as compared to that in the control (Fig. 5C).

### Inhibition of miR106b results in increased Rb gene expression in Huh7 cells

**Figure 6:**
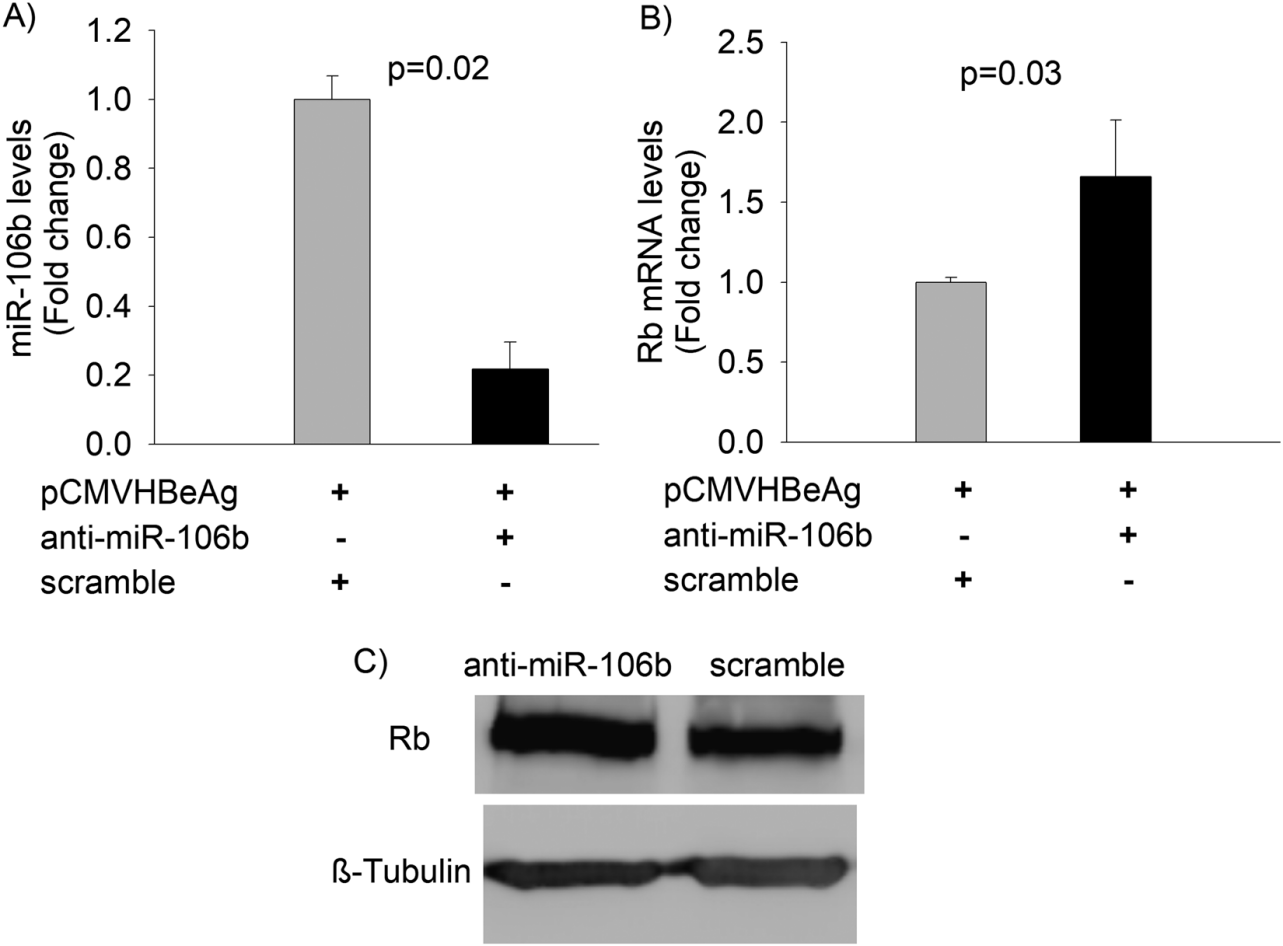
Quantitation of miR-106b and Rb expression levels following miR-106b inhibition. (**A**) Inhibition of miR-106b using anti-miR-106b oligonucleotides significantly decreased miR-106b expression in cells transfected with HBeAg-expression vector (pCMVHBeAg). (**B**) Inhibition of miR-106b expression using anti-miR-106b significantly increases Rb mRNA levels in Huh7 cells. (**C**) Western Blot analysis of pRb expression in the presence of anti-miR-106b or scramble in Huh7 cells (The full-length western blots images are shown in supplementary Fig. S3; the bar graphs are represented as mean ± SD with n = 3).

Our results confirm the link between transient expression of HBeAg and increased miR106b levels and reduced expression of Rb gene in Huh7 cells. The Rb gene has been experimentally validated as a target of miR-106b in laryngeal carcinoma cells^14^. We investigated the effect of anti-miR-106b on the expression of the Rb gene and on the proliferation of Huh7 cells. Anti-miR-106b inhibited miR-106b levels as expected (Fig. 6A). Anti-miR-106b-mediated reduction in miR-106b levels led to a significant increase in the Rb mRNA and protein levels (Fig. 6B and C).

### miR-106b increases proliferation and cell cycle progression in Huh7 cells by targeting the Rb gene

**Figure 7:**
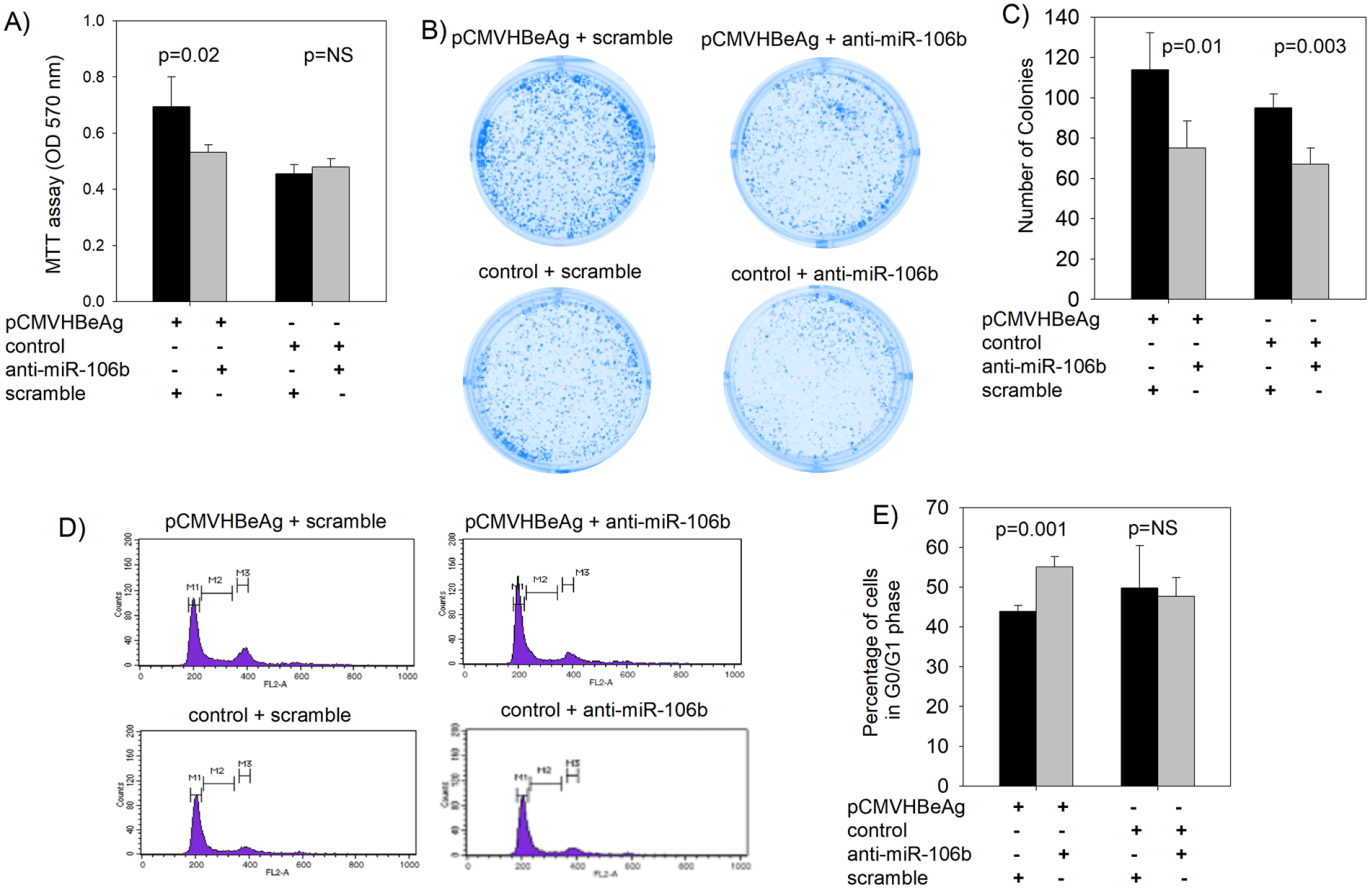
Cell proliferation and cell cycle analysis in the presence of anti-miR-106b. (**A**) Bar graphs showing anti-miR-106b-mediated inhibition of cell proliferation as assessed by MTT assay in Huh7 cells. (**B**) and (**C**) inhibition of colony formation by anti-miR-106b in Huh7 cells. (**D**) and (**E**) Flow cytometry analysis of cell cycle (The M1 peak corresponds to cells in G0/G1, M3 peak corresponds to cells in G2/M and cells in S phase are shown as M2) indicates that anti-miR-106b leads to the accumulation of increased proportion of cells in the G0/G1 phase in Huh7 cells expressing HBeAg (the bar graphs are represented as mean ± SD with n = 3; NS = not significant).

We evaluated the effect of loss-of-function of miR-106b on proliferation and cell cycle progression in Huh7 cells. Inhibition of miR-106b levels using anti-miR-106b in the cells co-transfected with the control did not affect cell proliferation (MTT assay, Fig. 7A) or the proportion of cells in the G0/G1 phase in the cell cycle (Fig. 7D and E). Whereas in the cells co-transfected with the HBeAg expression construct, anti-miR-106b led to a significant reduction in cell proliferation (MTT assay, Fig. 7A) and a significant increase in the proportion of cells in the G0/G1 phase (Fig. 7D and E). Anti-miR-106b inhibited colony formation regardless of the presence of HBeAg (Fig. 7B and C).

## Discussion

Chronic HBV infection is associated with HCC. The presence of HBeAg, a secretory protein of hepatitis B virus is an important determinant of disease sequelae associated with chronic hepatitis B infection. Importantly, HBeAg increases the risk of HCC independent of virus loads^15^. While the association between HBeAg and HCC has been known for decades, most studies on HBV-related HCC have focused on the HBx protein. The biological role of HBeAg remains poorly understood. Our results demonstrate that HBeAg is linked to increased cell proliferation (Fig. 1) and accelerated G1/S transition (Fig. 2). To the best of our knowledge, there are no reports investigating the role of HBeAg on cell proliferation or modulation of cell cycle. We believe that our study highlights a novel role of HBeAg in modulating cell proliferation and cell cycle progression; this will provide a new perspective to epidemiological studies linking HBeAg to HCC.

To understand the molecular mechanisms underlying the role of HBeAg in cell proliferation and cell cycle progression, we chose to investigate HBeAg-mediated dysregulation of host genes and host miRNAs. We believe that analysing changes in host gene /miRNA profiles may help understand the role of HBeAg in cell proliferation since a) It is becoming increasingly evident that aberrant expression of cellular miRNAs and their gene targets play a prominent role in modulating the pathogenesis of HBV-induced HCC^16–18^ b) HBx, an oncoprotein encoded by HBV is a major regulator of both gene expression and miRNA expression in the host^19–22^ and c) Recent reports indicate differences in host miRNA profiles between HBeAg positive and HBeAg negative chronic HBV patients^23,24^. Although studies have shown that the presence of HBV or HBV proteins, in general is linked to differential miRNA expression profile in HBV-infected hepatocytes and liver cancer cell lines^25–27^, the specific effect of HBeAg expression on host cells, including miRNA and mRNA expression profiles remains poorly understood.

Interestingly, the transient expression of HBeAg differentially regulated a total of 21 host genes and 12 host miRNAs (Figs 3 and 4). The number of genes and miRNA that are dysregulated by HBeAg were only a fraction of that reported for the HBx protein^28,29^. We then analysed, if any of the genes differentially regulated by HBeAg is an experimentally validated target of miRNAs that are dysregulated by HBeAg by integration of gene expression data and miRNA data. Interestingly, using this approach we identified that the Rb gene (a tumor suppressor gene down-regulated by HBeAg) is an experimentally validated target of miR-106b (a miRNA up-regulated by HBeAg). Lower levels of Rb protein (pRb) have been reported in patients with HBV-related HCC^30^.

MicroRNA-106b is a member of the miR-106b-25 cluster and is known to act as an oncogene that is associated with poor prognosis among HCC patients^31^. Previous studies have shown that miR-106b expression is significantly up-regulated in HCC cell lines and HCC tissues^32^. Of note, miR-106b expression has been shown to be affected by HBV infection^33^ but the underlying molecular mechanism(s) that regulates miR-106b expression in HBV-associated HCC is not well understood. The Rb gene is a tumor suppressor gene with a well-documented role in HCC including the modulation of cell proliferation and cell cycle progression^34,35^. We validated the microarray findings on the miR-106b and Rb gene using real-time PCR (Fig. 5A and B). In addition, HBeAg expression results in lower levels of pRb in Huh7 cells (Fig. 5C).

To further investigate the role of HBeAg-induced miR-106b, we used anti-miR-106b (anti-sense oligonucleotides that inhibit miR-106b). Reduction in miR-106b levels using anti-miR-106b led to an increase in Rb gene expression in Huh7 cells (Fig. 6), suggesting that the Rb gene is a target of miR-106b. A previous report has shown that the 3’UTR (3′ untranslated region) of the Rb gene harbors a mir-106b binding site^14^.

Anti-miR-106b inhibited cell proliferation in HBeAg expressing Huh7 cells but not in control Huh7 cells as measured by the MTT assay and flow cytometry analysis (Fig. 7); these findings clearly highlight the link between HBeAg-induced miR-106b and enhanced cell proliferation of Huh7 cells.

Colony formation in Huh7 cells was significantly inhibited by anti-miR106b regardless of HBeAg expression (Fig. 7B and C). Differences in cell proliferation analysis using MTT assay, colony formation assay and flow cytometry for the same experimental conditions have been reported previously^36,37^; the differences in experimental time (MTT vs colony formation) has been suggested as a possible reason.

Our results are in keeping with a previous report on laryngeal carcinomas, in which miR-106b was shown to target the Rb gene resulting in increased cell proliferation and cell cycle progression^14^. Interestingly, a very recent report has identified that miR-106b is over expressed in HBV-related HCCs as compared to HCV-related HCCs or non-B/non-C-associated HCCs^38^. Overall, our results demonstrate that HBeAg induces miR-106b that targets the Rb gene, leading to enhanced cell proliferation and promoting progression from G0/G1 phase into S phase of cell cycle in Huh7 cells. A limitation of our study is that all the findings pertain to hepatoma cells; it may be interesting to perform these experiments in primary liver cells.

**Figure 8:**
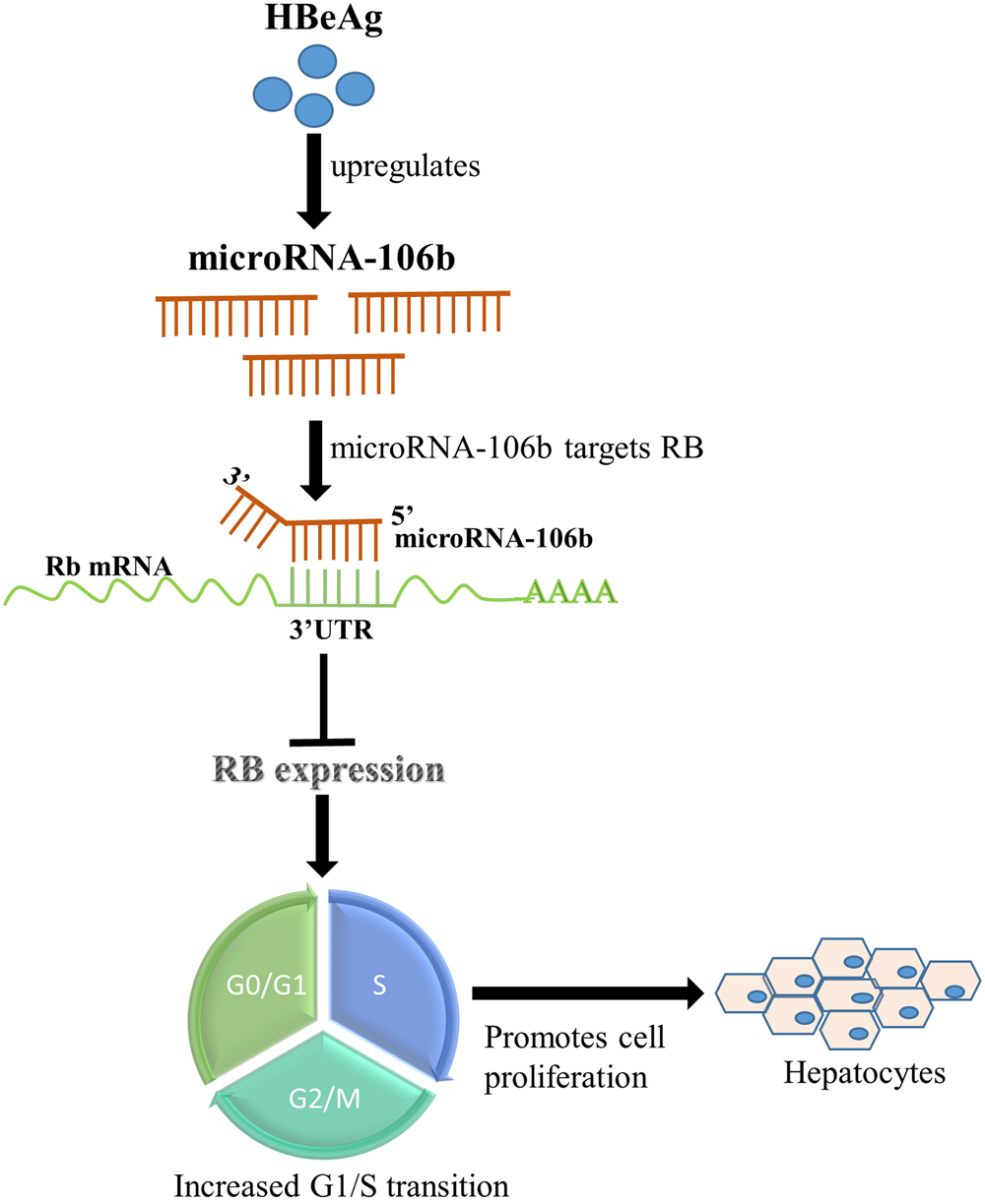
A schematic diagram illustrating a role for HBeAg in the pathogenesis of HBV-related hepatocellular carcinoma.

A schematic illustrating our finding on the association between HBeAg and HBV-related HCC is shown in Fig. 8. To our knowledge, this is the first report identifying a possible biological role for HBeAg in progression to HCC. Our findings suggest that HBeAg is an oncoprotein that inhibits the Rb gene expression via induction of an oncogenic miRNA, miR-106b.

In our study we did not specifically address how HBeAg induces miR-106b. Several reports indicate virus-mediated dysregulation of host miRNA^39,40^. Nonetheless, the specific mechanisms of virus /virus protein-mediated dysregulation of miRNA remain elusive. It has been suggested that dysregulation of miRNA may occur via modulation of primary miRNA transcription or through mechanisms that affect processing of pri-miRNA or pre-miRNA^40^. Down-regulation of host miRNA by antisense RNA-mediated degradation is reported among herpesviruses^41^. Upregulation of host miRNA by enhancing primary miRNA transcription is reported for Influenza A virus^40^; nonetheless specific mechanisms of virus protein-mediated induction of host miRNA remain unclear.

In summary, our findings highlight a role for HBeAg in HBV-related HCCs. First, we show that HBeAg induces cell proliferation and accelerates progression of cells from the G0/G1 phase to the S phase of the cell cycle. We then identified a total of 21 host genes and 12 host miRNA dysregulated by HBeAg. Specifically, HBeAg-induced miR-106b targets the Rb gene, a well-known tumor suppressor gene. Finally, the inhibition of HBeAg-induced miR-106b increased Rb gene expression resulting in reduced cell proliferation and slowing of cell cycle progression by arresting cells in the G0/G1 phase. Our results suggest a possible role for HBeAg in HBV-related HCCs.

## Methods

### Plasmids and constructs

The complete HBV pre-C/C ORF (nt. 1814 to 2452) was amplified and cloned into a mammalian expression vector (pEGFP-n1) under the control of the CMV (cytomegalovirus) promoter (pCMVHBeAg). We then introduced the G1896A mutation in pCMVHBeAg to create a premature stop codon rendering this construct incapable of HBeAg synthesis; this plasmid is referred to as the control plasmid. The production of HBeAg by pCMVHBeAg was ascertained by testing the culture supernatant in a commercially available ELISA (enzyme-linked immunosorbent assay) kit (HBeAg kit, Dia.Pro; data not shown). The HBx-expression construct and the HBV surface-expression construct were created as described previously^17,42^.

### Cell culture

Huh7 (human hepatoma) cells were cultured in Dulbecco,s modified eagle medium (DMEM, gibco, Thermo Fisher Scientific) supplemented with 10% fetal bovine serum (FBS, gibco, Thermo Fisher Scientific), 5000U/ml penicillin and 5000 µg/ml streptomycin (Penicillin-Streptomycin, gibco, Thermo Fisher Scientific) and maintained at 37 °C with 5% CO_2_. The pCMVHBeAg (HBeAg expressing) or the control plasmid (a plasmid that contains a pre-mature stop codon, does not express HBeAg) were transfected into Huh7 cells. Cells and supernatants were harvested 48 hours post-transfection for RNA (ribonucleic acid) and protein analyses. All transfections were done in triplicate.

A miRNA inhibitor for miR-106b (anti-miR-106b, Cat. No. MIN0000680, Qiagen) and a non-specific control sequence (scramble; used as negative control, Cat. No. 1027271, Qiagen) were purchased from Qiagen. For inhibition of miR-106b expression, miR-106b inhibitor (50 nM) or negative control (50 nM) was co-transfected with pCMVHBeAg in Huh7 cells using lipofectamine2000 (Cat. No. 11668019, Thermo Fisher Scientific) as described previously^14^. Cells were harvested 48 hours post-transfection for analysis.

### MTT assay

Huh7 cells were seeded at a density of 1 × 10^4^ cells per well in a 24-well plate. Forty eight hours post transfection, fresh media containing 0.1 mg/ml MTT (Cat No. TC191, Himedia) was added to cells and the plates were incubated for 30 min at 37 °C. After removing the medium, 300 µl dimethyl sulfoxide (DMSO, Cat. No. 24075, SRL Pvt. Ltd.) was added to each well to solubilise the purple formazan crystals. The plates were analysed by measuring the optical density at 570 nm.

### Colony Formation Assay

Twenty-four hours post transfection, cells were trypsinized and seeded into a fresh 6-well plate at a density of 2000 cells per well. Cells were allowed to grow and form visible colonies for 8–10 days with change of media on alternate days. Colonies were fixed with ethanol, stained with 0.5% crystal violet (0.5% crystal violet made in 25% methanol) (Crystal violet, Cat. No. C3886, Sigma-Aldrich) and visualized and counted using light microscope as described previously^42^.

### Cell cycle analysis

For cell cycle analysis, twenty four hours post-transfection, cells were synchronized using serum-free medium (DMEM with 0.2% FBS) for 12 hours and then were stimulated by replacment with complete growth medium for 24 hours. Then, cells were washed, trypsinized, fixed with 70% ethanol and treated with 5 μl RNAse A (10 mg/ml, Cat. No. EN0531, Thermo Fisher Scientific) and stained using 50 μg/ml propidium iodide (Cat. No. P4170, Sigma-Aldrich) for 30 minutes at 37 °C^43^ and analysed using BD FACS Calibur flow cytometer (BD Biosciences). The cell cycle kinetics were analysed using Cell quest software (BD Biosciences).

### cDNA microarray profiling and data analysis

To determine the differential gene expression profiles, Huh7 cells were transfected with HBeAg-expression construct (pCMVHBeAg) or the control; we then performed expression profiling using affymetrix primeview^TM^ human gene expression genechips. Total RNA was extracted from cells using TRIzol reagent (Cat. No. 15596018, Thermo Fisher Scientific) according to manufacturer’s instructions. cDNA synthesis, *in vitro*-transcription and labelling of RNA was done using the Gene chip IVT express kit (Affymetrix, Thermo Fisher Scientific). Hybridization of labelled RNA to primeview^TM^ human gene expression arrays was done followed by staining and washing using Gene chip fluidics station and the arrays were scanned using Gene Chip® Scanner (Affymetrix). cDNA from two biological replicates were tested separately in the cDNA microarray. The mRNA expression data were analyzed using genespring software GX 12.1. A fold change of 1.5 times with a p value < 0.05 was used to identify differentially expressed genes. The fold change differences are presented in terms of log base 2. The RMA algorithm (robust multiarray average algorithm) was used for statistical analysis. The intra array normalization was done using quantile normalization and inter-array normalization was done by calculating median of all samples.

### MicroRNA microarray profiling and data analysis

To assess the host cellular miRNA expression profile in response to HBeAg expression in Huh7 cells, we analyzed differential miRNA expression profile using affymetrix genechip miRNA 2.0 arrays. Total RNA was extracted from cells using TRIzol reagent according to manufacturer’s instructions. Poly A tailing and labelling of RNA was done using flash tag HSR Biotin labelling kit (Affymetrix, Thermo Fisher Scientific) followed by hybridization of biotin labelled samples to the arrays. The arrays were then stained and washed using Gene chip fluidics station and scanned using Gene Chip® Scanner. Two biological replicates were tested separately for the miRNA microarray. The miRNA expression data were analyzed using genespring software GX 12.1. A fold change of 2.0 times with a p value < 0.05 was used to identify differentially expressed miRNAs. Data analysis was done using the same methods described for cDNA microarrays.

### Real time PCR

Total RNA was extracted from cells using TRIzol as per the manufacturer’s instructions. The RNA pellet was dissolved in nuclease-free water (Cat. No. 129114, Qiagen). cDNA was synthesized from 1 µg of DNase I (Cat. No. M0303, New England Biolabs) treated RNA using the iScript cDNA synthesis Kit (Cat. No. 1708891, Bio-rad). For quantitation of microRNA (miRNA) expression levels, stem-loop RT PCR (real-time polymerase chain reaction) was performed. cDNA was synthesized using specific stem-loop miRNA-specific primers. U6 snRNA was used as an internal control for quantitation of expression levels of miRNAs. Real time PCR was done using miRNA-sequence specific forward primer and a common stem loop reverse universal primer for all miRNAs and U6 snRNA (For the sequences of primers, see Supplementary Table S1).

### Western blots

Total protein was extracted from transfected cells using RIPA lysis buffer (Pierce RIPA buffer, Cat. No. 89900, Thermo Fisher Scientific) and protease inhibitor (Pierce protease inhibitor, Cat. No. 88266, Thermo Fisher Scientific) as per the manufacturer’s instructions. For western blot analysis, equal amounts of protein were separated on 10% SDS (sodium dodecyl sulfate) gel and transferred to nitrocellulose membrane (Cat. No. RPN68D, GE healthcare Life Sciences). After the transfer, the membrane was blocked with 5% BSA (Bovine serum albumin, Cat. No. MB083, Himedia) followed by incubation with antibodies specific for mouse anti-Rb (1:300 dilution, Cat. No 554136, BD pharmingen) and mouse anti-tubulin (1:10,000, Cat. No. sc-8035, Santa Cruz Biotechnology) for overnight. After washing, blots were incubated with goat anti-mouse HRP-conjugated secondary antibodies (Cat. No. sc-2005, Santa Cruz Biotechnology) and visualized using enhanced chemiluminescence (Pierce ECL plus, Cat. No. 32132, Thermo Fischer Scientific). The β-tubulin signal was used as a loading control.

### Data analysi**s**

All the data were analyzed using student’s *t* test. Graphs were made using the software Sigma plot. Results were considered statistically significant at a P value < 0.05. All data are presented as mean ± SD.

Our study does not have any experiments involving human participants or animals.

## Electronic supplementary material

Supplementary information
