# Supplementary material for: HBeAg-induced miR-106b promotes cell growth by targeting the retinoblastoma gene
Source: Sci Rep. 2017 Oct 30;7:14371. doi: 10.1038/s41598-017-14652-x (PMC5662563; doi:10.1038/s41598-017-14652-x)
Supplement: Supplementary file 1 — Supplementary information [file 41598_2017_14652_MOESM1_ESM.pdf]

Title of manuscript

HBeAg-induced miR-106b promotes cell growth by targeting the retinoblastoma gene

Authors

Jasmine Samal, Manish Kandpal and Perumal Vivekanandan

Supplementary Table S1: **Sequences of primers and inhibitor used in the study**

S.No	PRIMER NAME	SEQUENCE
1	miR-106b stem loop cDNA specific	5’GTCGTATCCAGTGCAGGGTCCGAGGTATTCGCACTGGATACG ACATCTGC 3’
2	miR-106b qPCR F	5’ GACGCTAAAGTGCTGACAGT 3’
3	miR-106b qPCR R	5' GTGCAGGGTCCGAGGT 3'
4	RB qPCR F	5’ AGGATCAGATGAAGCAGATGG 3’
5	RB qPCR R	5’ TGCATTCGTGTTCGAGTAGAAG 3’
6	snU6 stem loop cDNA specific	5’GTCGTATCCAGTGCAGGGTCCGAGGTATTCGCACTGGATACG ACAAAATATGGAAC 3’
7	snU6 qPCR F	5’ GCCCCTGCGCAAGGATGAC 3’
8	snU6 qPCR R	5' GTGCAGGGTCCGAGGT 3'
9	GAPDH qPCR F	5’ TGCACCACCAACTGCTTAGC 3’
10	GAPDH qPCR R	5’ GGCATGGACTGTGGTCATGAG 3’
11	Anti-miR-106b inhibitor	5'UAAAGUGCUGACAGUGCAGAU3’

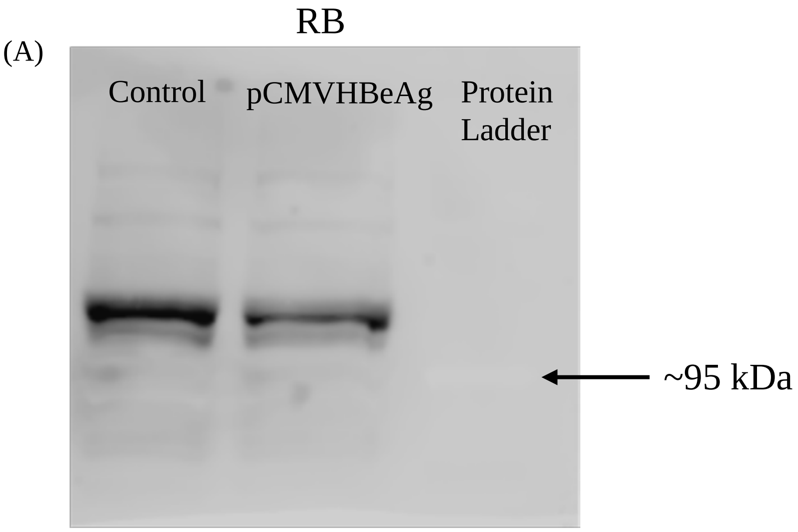

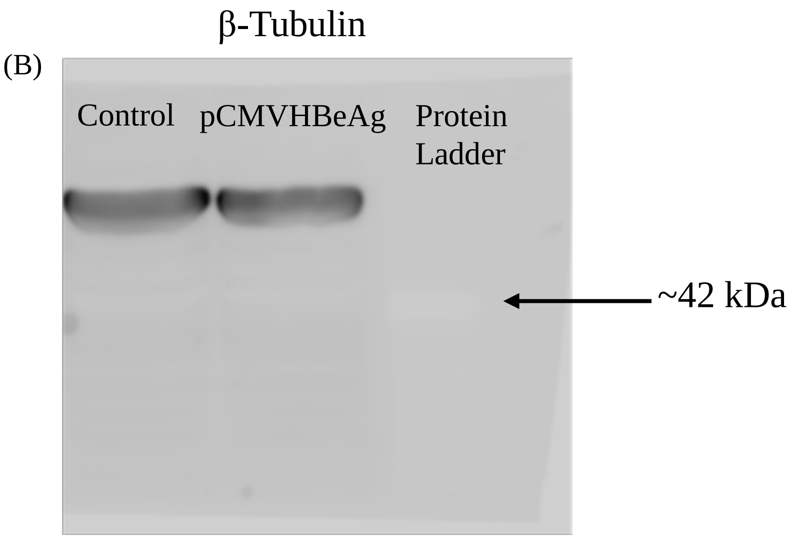

Supplementary Fig. S2: The full length Western blotting images for the cropped images shown in Fig. 5C. The nitrocellulose membrane was cut into two halves and the upper half of the membrane was probed with RB antibody and the lower half was probed with β-Tubulin antibody. The protein ladder used has a range of 10-175 kDa.

RB

(A)

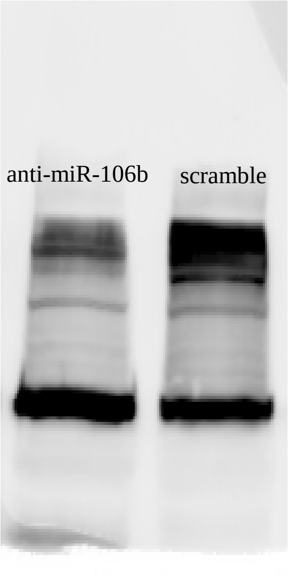

β-Tubulin

(B)

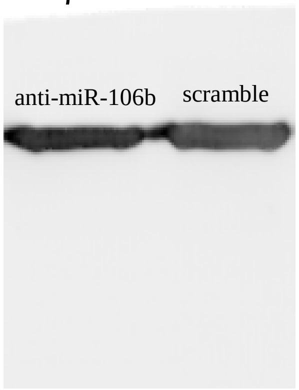

Supplementary Fig. S3: The full length Western blotting images for the cropped images shown in Fig. 6C. The nitrocellulose membrane was cut into two halves and the upper half of the membrane was probed with RB antibody and the lower half was probed with β-Tubulin antibody

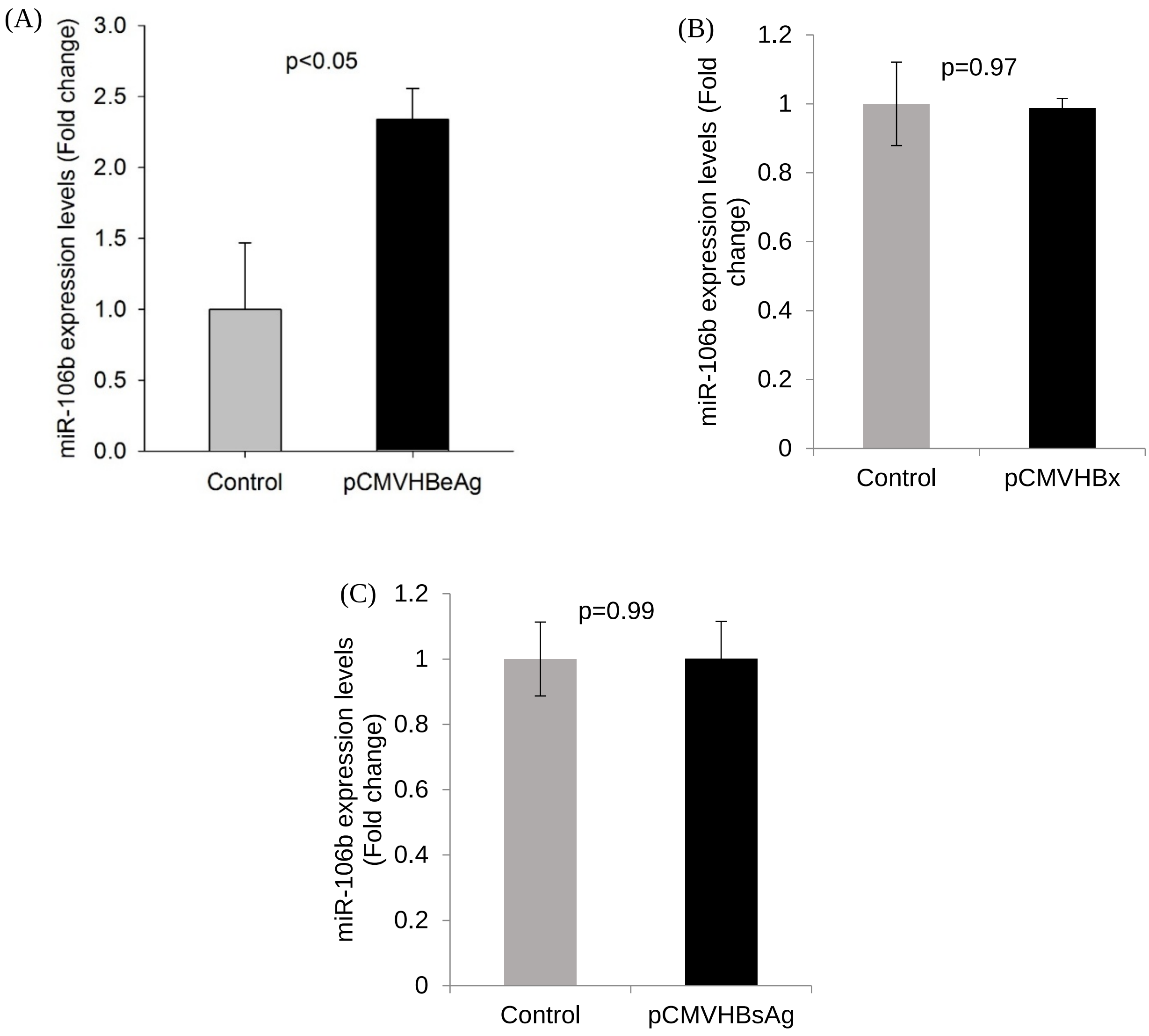

Supplementary Fig. S4: Quantitation of miR-106b expression levels using real time PCR. (A) HBeAg induced miR-106b levels in HepG2 cells. The HBV proteins (B) HBx and (C) HBsAg did not induce miR-106b expression in Huh7 cells.
